# PROTACs in cancer immunotherapy: a minireview

**DOI:** 10.1042/BST20253065

**Published:** 2025-10-01

**Authors:** Köckenberger J. E., Cardenas Alcoser E. S., Chang E. T., Gutkind J. S., Ferguson F. M.

**Affiliations:** 1 Department of Chemistry and Biochemistry, University of California San Diego, La Jolla, CA, 92093, U.S.A.; 2 Moores Cancer Center, University of California San Diego Health, La Jolla, CA, 92037, U.S.A.; 3 Department of Pharmacology, University of California San Diego, School of Medicine, La Jolla, CA, 92093, U.S.A.; 4 Skaggs School of Pharmacy and Pharmaceutical Sciences, University of California San Diego, La Jolla, CA, 92093, U.S.A.

**Keywords:** cancer, immune response, ubiquitin ligases, ubiquitin proteasome system, ubiquitin signaling

## Abstract

The discovery of immune checkpoint blockade as a therapeutic strategy to induce immunogenic cancer cell elimination has shown great success in the treatment of various cancers. However, limited response rates highlight the need for further development in this field. Promising new preclinical developments include the discoveries of proteolysis-targeting chimeras (PROTACs) to interfere with tumor immune escape signaling. Pharmacological induction of targeted protein degradation by these chimeras has shown advantages in inhibiting non-enzymatic protein functions and difficult to target protein–protein interactions. Furthermore, the induced degradation was shown to promote changes in the major histocompatibility complex I ligandome, which can be leveraged for an immune stimulus, increasing the cancer immune response. In this minireview, we highlight the research efforts ongoing towards employing PROTACs in immunotherapy for cancer treatment. Specifically, we outline how the unique mechanism of action can be leveraged to enhance the immune response or inhibit immune suppression.

## Introduction

The development of immune checkpoint blockade (ICB) therapies has revolutionized the landscape of oncological treatment options. Immune checkpoint proteins are frequently expressed by antigen-presenting cells and prevent the development of autoimmunity by limiting the excessive activation of lymphocytes, such as CD8^+^ T cells, by binding to cognate receptors on these immune cells. Cancers exploit this signaling pathway by expressing immune checkpoint ligands (e.g. programmed death ligand 1, PD-L1). PD-L1 binds its cognate receptor programmed cell death protein 1 (PD-1), expressed in activated CD8^+^ T cells, to prevent cancer cell targeting by the immune system. Blocking these immune checkpoints with therapeutic antibodies, for example, anti-PD-1 or anti-PD-L1, inhibits this immune suppression process and facilitates CD8^+^ T cell-induced tumor cell killing [1,2]. Based on this approach, eight monoclonal antibodies were approved by the FDA to treat various solid tumors, including melanoma, Hodgkin’s disease, non-small cell lung cancer, and renal cell carcinoma [3,4]. For example, in melanoma patients, the response to ICB treatment has increased the progression-free and overall survival rates, and the effects are more durable than with targeted agents, such as specific BRAF and MEK inhibitors, often achieving complete cancer remission [5–8]. However, primary and acquired resistance restricts the efficacy of ICB. For example, the non-response rate in melanoma is approximately 60% [3,9], and this is even higher in multiple other solid tumors like uveal melanoma at about 88% [10]. The mechanisms and reasons for ICB therapy failure are complex and not fully understood yet. Some studies show that change of immunopeptidome in the tumor environment correlates with higher efficacy in anti-CTLA-4 treatment [11]. Others correlate mutations of signaling components like JAK1/2 [12] and antigen presentation machinery, like B2M [12,13], or aberrant Gαs signaling and T cell exhaustion [14] to ICB therapy failure. Generally, combination therapy (anti-PD-L1 and anti-CTLA-4) has proven to be more effective, inhibiting multiple immune checkpoints at once [3,7,15]. This data demonstrates the potential of ICB but also highlights the need for further development of immunotherapy options for non-responding patients.

To address these challenges, it is now possible to use small molecules, even in difficult-to-target protein–protein interactions. Specifically, targeted protein degradation is a new modality in drug discovery research to target signaling processes previously considered undruggable. In this approach, compounds hijack the ubiquitin-proteasome system to deplete the protein of interest (POI). At present, the two major groups of target protein degrading drugs are bivalent molecules called proteolysis-targeting chimeras (PROTACs) or monovalent molecules called molecular glues. Their mechanism of action is to induce and stabilize a ternary complex of a POI with an E3-ligase by binding at their interface. Formation of such a ternary complex leads to the ubiquitination and proteasomal degradation of the POI. The stability of ternary complexes is a key factor for protein degradation efficiency. It is mainly influenced by co-operativity based on protein–protein interactions as well as ligand dissociation kinetics. The latter can be manipulated by incorporating electrophilic warheads to render ligand binding irreversible and has found recent attention. However, due to the event-driven mechanism of action, slow off rates might also render the protein turnover inefficient [16,17]. So far, more than 25 PROTACs have reached clinical trials in a variety of cancer types, including prostate cancer and non-small cell lung cancer, and at least one is already under evaluation in a Phase III trial in ER+/HER2− breast cancer [18,19].

**Figure 1:**
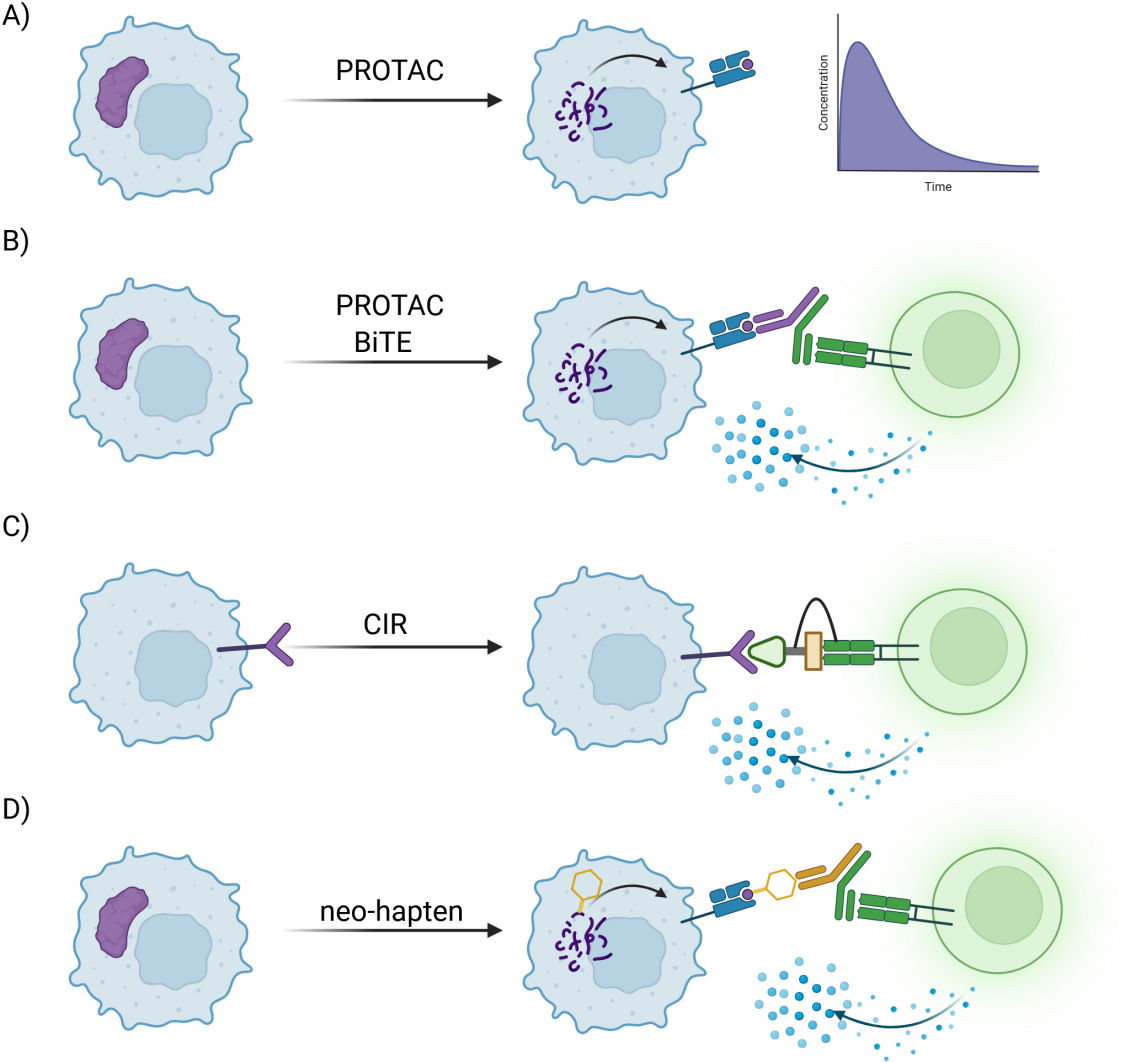
PROTACs and covalent strategies can introduce neoantigens targetable with antibodies. (**A**) Jensen et al. [20] and Moser et al. [21] find degradation products enhanced presentation on MHC-I but these effects are too transient to induce T cell activation alone. (**B**) Massafara et al. [22] target degradation products by using bifunctional antibodies to recruit and activate T cells. (**C**) Covalent immune recruiters (CIR) directly recruit and activate T cells by forming covalent ternary complexes on their cell surfaces [23–25]. (**D**) Covalent ligands can form neo-haptens which are targetable by bispecific antibodies [26,27]. Figure created with Biorender.

With this work, we highlight how due to their unique mechanism of action PROTACs deplete enzymatic and non-enzymatic roles of immune suppressive proteins and influence the antigen presentation by augmenting intracellular peptide concentrations. Hence, they are addressing key immunotherapy challenges like lack of neoantigen presentation and increased immune suppression (Figure 1).

## Increased and guided immune responses

T-lymphocytes recognize peptide antigens presented by cancer cells on the major histocompatibility complex I (MHC-I) via T cell receptors (TCRs). Once activated, CD8^+^ T cells will induce tumor cell killing and secretion of cytokines such as interferon γ (IFNγ) and tumor necrosis factor α [28,29]. These cytokines will activate other immune cells and enhance T cell migration, further enhancing the tumor cell killing. The peptides presented on the MHC-I are generally derived from self-protein or defective ribosomal products (DRiPs) degradation [30–32]. After ubiquitin and proteasome-dependent degradation, the peptides are transported to the endoplasmic reticulum by the transporter for antigen processing, where they are further processed and loaded on the MHC-I [33–35]. Targeted protein degraders not only produce but also enhance neo-peptide abundances by induced degradation, leaving the question of whether this part of the mechanism could be leveraged in immunotherapy [20–22].

To tackle this question, Jensen et al. [20] studied the peptide abundance on the MHC-I by LC-MS/MS following the treatment with PROTAC degrading BET-family proteins. They identified four peptides linked to BRD3 and BRD4 increasingly presented on the MHC-I when the cells were treated with degrader compounds. The timeline of enhanced presentation matched the degradation kinetics, tying the enhanced presentation to the induced degradation. A further highly interesting finding is that while the same four peptides were detected for CRBN- and VHL-dependent degradation, only two of these peptides were detected when treating with the MDM-2 recruiting PROTAC. This study shows that not only is the MHC-I ligandome highly influenced by proteasome-dependent degradation, but also by the E3 ligase involved. However, no general enhancement of MHC-I on the cell surface was detected, rendering an immune response based on this effect unlikely (Figure 1A).

Moser et al. [21] developed a model antigen construct (GFP-S8L-F12), which can be degraded using the dTAG system previously described in literature [36]. In this system, FKBP12^F36V^ fusion proteins can be degraded with PROTACs (dTAG compounds) composed of Ortho AP linked to either a CRBN or VHL recruiter [36]. The generated peptide antigen and its presentation on the MHC-I were traced using antibodies and flow cytometry studies. Similar findings to Jensen et al. [20] were made, showing that the contribution of DRiPs was lower than proteasome-dependent antigens to the presentation on MHC-I, which is further strongly influenced by the degradation kinetics. However, the authors point out that the effect is transient since the protein abundance is quickly lowered, which in turn will lead to lower antigen abundance and presentation. These findings again show that specific peptides can be targeted for MHC-I presentation using targeted protein degradation. However, in contrast with regular PROTAC optimization, to increase an immune response, a slower and steadier degradation kinetic is desirable (Figure 1A).

To address these challenges, Massafra et al. [22] combined the use of PROTACs with T cell bispecific antibodies. These T cell bispecific antibodies recognize a specific peptide derived from Wilms tumor protein 1 (WT1) and further harbor a TCR binding/recognition site. In their study, they found that combining a WT1-degrading PROTAC with a bispecific antibody enhanced T cell activation markers. Further, enhanced tumor cell killing in a primary T cell coculture with cancer cells was found when cells were pretreated with the WT1-degrading PROTAC (Figure 1B).

Aside from PROTACs, covalent inhibitors were found to influence the immunopeptidome, as covalently modified peptides are presented as a neo-hapten, inducing cell death to resistant cancer cells or offering novel antibody targets [26,27]. However, novel peptides induced by PROTACs or covalent inhibitors face the challenge of transient presentation, increasing the need for very high affinity immune complexes to induce productive ‘cell–cell’ proximity. Stabilization of immune complexes has been achieved by Serniuck et al. and Kapcan et al. using electrophilic warheads to covalently link a bifunctional molecule to antibodies or immune cell receptors, recruiting them to surface proteins on cancer cells [23–25] (Figure 1C and D).

The general advantage of such strategies is that any protein specific to any cancer could be targeted for degradation since ultimately CD8^+^ T cells would facilitate cancer cell killing. This would enable cancer-specific non-essential proteins to be leveraged for immune system activation. Combining PROTACs with ICB therapy might overcome the lack of neoantigens. The combination of PROTACs with a covalent immune recruiter strategy could enable targeting of non-essential proteins for degradation to facilitate immune tumor lysis through induced cell–cell proximity based on the targeted protein degradation peptide products.

## Overcoming tumor immune suppression

Due to evolutionary pressure, many cancers have developed different strategies to undermine the function of the immune system, which would usually kill mutated dysregulated growing cells. Many such mechanisms include the expression of immune suppressive proteins. These proteins include PD-L1 and immune suppressive pathway components, such as indoleamine 2,3-dioxygenase 1 (IDO-1) or cyclooxygenase-1/2 (COX-1/2). Their effect varies from direct inactivation of CD8^+^ T cells (PD-L1) to enhanced concentration of metabolites, such as kynurenine (IDO-1) or prostaglandin E_2_ (PGE_2_) (COX-1/2), which cause immune suppression. However, these proteins can be tough to target pharmacologically since they function through protein–protein interactions (PD-1/PD-L1) or their immune suppressive role extends beyond the enzymatic function (e.g. IDO-1 [37]). To overcome these signaling and immunosuppressive roles, targeted protein degradation can be leveraged.

### PD-L1-PROTACs

**Figure 2:**
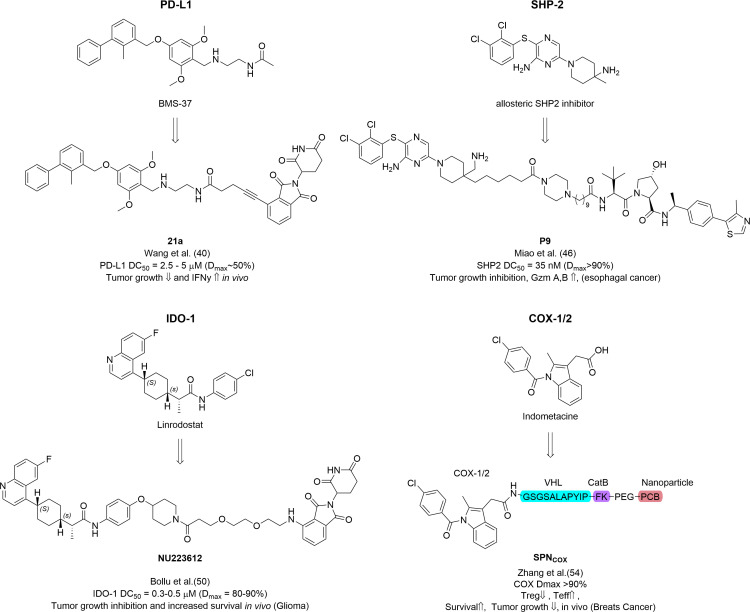
Chemical structures of PROTACs to overcome tumor immune escape by targeted degradation of immunosuppressive protein.COX-1/2, cyclooxygenase-1/2; IDO-1, indoleamine 2,3-dioxygenase 1; PD-L1, programmed death ligand 1.

In general, PD-L1 is a challenging target for PROTACs since only very few small molecule PD-L1 binders are known [38]. Cheng et al. [39] linked the PD-L1 binders BMS-8 or BMS-1198 to lenalidomide [38,39]. After linker optimization efforts, the authors found that compound P22 showed the most efficient inhibition of PD-1/PD-L1 interaction (IC_50_=39.2 nM) but only minor degradation of PD-L1 (14% by flow cytometry and 35% by Western blot at 10 µM). This degradation was rescued by bafilomycin, indicating a lysosomal degradation. However, the minor degradation of PD-L1 by P22 and a strong PD-L1 enrichment by bafilomycin treatment alone restrict the conclusiveness of this experiment. Functionally, P22 restored interferon γ (IFN γ) secretion in a T cell co-culture experiment with slightly better efficacy than the anti-PD-1 antibody pembrolizumab. Wang et al. [40] further optimized PROTACs targeting PD-L1 and highlighted their potential by discovering compound 21a based on the PD-L1 binder BMS-37 and a thalidomide analogue joined by a short linker (Figure 2). Compound 21a was found to degrade more than 50% PD-L1 at 5 µM in MC-38 cells, a colon adenocarcinoma cell line with high expression levels of PD-L1. Compound 21a was further tested in seven other cell lines, showing the best degradation in SKNO-1 (leukemia) and PC-3 (prostate cancer) cells. The authors confirmed, in contrast with P22, that the degradation effect of 21a is rescued by proteasome and NAE inhibition, as well as CRBN and PD-L1 binding competition. Since PD-L1 is a membrane-bound protein and the binding site for BMS-37 is located on the extracellular part, the authors argue that induced degradation of PD-L1 must happen with internalized and newly synthesized PD-L1. To test this hypothesis, confocal microscopy was used to validate that mainly cytosolic PD-L1 is degraded. Finally, 21a was further proven to inhibit tumor growth, enhance gene expression levels of immune markers such as IFNγ and improve intratumoral CD8^+^ T cell invasion *in vivo*. Liu et al. [41] expanded the scope of PD-L1 degraders, including recruitment of different E3 ligases (VHL and MDM2) in combination with different PD-L1 binders, using a combinatorial copper-assisted alkyne-azide cycloaddition (CuAAC) approach. Even though some activity was shown for the VH032-linked BMS-37 PROTACs, the thalidomide-conjugated BMS-37 analogs (e.g. BMS-37-C3) similar to P22 or 21a were the most efficient degraders (-50% PD-L1 at 3 μM).

These studies prove the promising benefits of targeting PD-L1 with a PROTAC approach, with promising immunostimulatory data *in vitro* and *in vivo*. However, a highly effective chemical probe matching the criteria of DC_50 _< 1 μM, D_max _> 80% and unbiased proteomics off-target profiling is yet to be published.

### SHP-2 PROTACs

Src homology region 2 domain-containing phosphatase-2 (SHP-2) acts downstream of many receptor tyrosine kinases and is involved in MAPK-ERK activation. SHP-2 was found to be central to PD-1 intracellular signal transduction causing immunosuppressive effects. Therefore, SHP-2 inhibition or degradation is a potential therapeutic strategy for cancer immunotherapy in combination with other immunomodulatory drugs. The SHP-2 PROTAC SHP2-D26 was discovered by Wang et al. [42] linking a known allosteric SHP2 inhibitor (SHP099) to VH032. SHP-D26 showed strong degradation potency and efficacy, with DC_50_ values of 6.0 and 2.6 nM in esophageal cancer (KYSE-520) and acute myeloid leukemia (MV-4–11) cells, and a D_max_ of >95%. The degradation of SHP-2 led to enhanced inhibition of ERK phosphorylation and cell growth compared with the parental allosteric inhibitor SHP099. Vemulapalli et al. [43] discovered the SHP2 degrader R1-5C by linking RMC-4550 to a thalidomide analogue. R1-5C was evaluated for its activity by western blotting (<−90% at 10 nM) and its selectivity by proteomics (no off-targets at 4 h and 8 h incubation) in MV-4–11 cells. However, the authors did not find any benefit of SHP-2 degradation over inhibition in growth suppression of esophageal cancer (KYSE-520) and myeloid leukemia (MV-4–11) cells. Zheng et al. [44] combined SHP099 with pomalidomide via different PEG-linkers using CuAAC leading to the discovery of SP4. SP4 induced SHP2 degradation in HeLa cells at 4.6 nM after 24 h (~−50%) and 48 h (~−70%). SP4 was found to induce apoptosis and inhibit HeLa cell growth (IC_50_=4.3 nM) better than the parental inhibitor SHP099 (IC_50_=566 nM). Finally, Yang et al. [45] introduced compound 11 (DC_50_=6.02 nM in MV-4–11), composed of TNO155, a SHP2 allosteric inhibitor analogue, and pomalidomide, joined by a PEG3-linker. Compound 11 was also found to decrease cell viability of leukemia MV-4–11 cells. PROTAC P9, developed by Miao et al. [46] in 2023, degraded SHP-2 with a DC_50_ of 35.2 ± 1.5 nM and showed remarkable *in vivo* efficacy with almost complete tumor regression in a KYSE520 esophageal cancer xenograft mouse model while being well tolerated over 18 days (Figure 2). While these remarkable effects in cancer cell growth and survival can be explained by the ability of SHP2 PROTACs inhibiting cancer signaling, such as ERK, an exciting possibility exists to evaluate their potential to increase ICB response.

### IDO-1-PROTACs

The IDO-1 enzyme converts tryptophan to downstream metabolites called kynurenines. Since the discovery of IDO-1 overexpression and its immunosuppressive role in different cancers, inhibitors have been developed. However, the inhibitors failed in clinical trials due to a lack of efficacy [47,48]. Further studies have indicated that non-enzymatic functions of IDO-1 involving up-regulation of expression levels (e.g. complement factor H) may be the reason for therapy failure, especially in glioblastoma treatment [37]. Targeted protein degradation is an ideal alternative approach to target and investigate these non-enzymatic effects of IDO-1 in tumor immune escape. PROTACs to degrade IDO-1 by Hu et al. [49] were based on the potent inhibitor epacadostat linked to thalidomide using various length PEG linkers, which identified the compound 2c (PEG_8_) as a moderately potent but effective IDO-1 degrader (DC_50_=2.5 µM, D_max_ 93%). However, when excessive IFN-γ is used to induce more overexpression of IDO-1, the degraders’ capacity of degradation was exceeded. Further, 2c was investigated in an HER2 CAR-T cell coculture model, moderately enhancing the tumor-killing activity. Bollu et al. [50] investigated IDO-1 degradation in glioblastoma models. To design this PROTAC, an analogue of the IDO-1 inhibitor linrodostat (BMS-986205) was linked to either VH032 or thalidomide analogs via aromatic ether formation. Their efforts led to the discovery of NU223612, a potent degrader of IDO-1 in glioblastoma cells (DC_50_=0.3–0.5 µM, Figure 2). Mechanistically, the CRBN and proteasome dependency of NU223612-induced degradation was proven by rescue experiments as well as negative control probes. NU223612 was shown to reduce non-enzymatic functions of IDO-1, such as NF-κB and p65 phosphorylation. Finally, the PROTAC was found to be blood-brain-barrier penetrative, to effectively reduce IDO-1 concentrations and to lead to a survival benefit over vehicle control in glioma mouse models. In summary, these studies highlight IDO-1 as a promising target for PROTAC development to overcome immune suppression in IDO-1 overexpressing cancers.

## COX-1/2 PROTACs

COX enzyme acts on the arachidonic acid pathway, catalyzing the biosynthesis of prostanoids like PGE_2_, and exists in two distinct isoforms, COX-1 and COX-2. While COX-1 serves essential functions such as in hemostasis, COX-2 is associated with pain, inflammation, and renal function. In various animal models of colon, lung, and breast cancer, inhibition or genetic deletion of COX-2 was found to reduce tumor burden [51,52]. PGE_2_ was found to promote immunosuppressive cells like regulatory T cells or M2-type macrophages [53] and is further involved in CD8^+^ T cell exhaustion [14]. To target COX-1/2 specifically in the tumor microenvironment, while preserving its essential functions in the rest of the organism, Zhang et al. [54] developed a smart nano-PROTAC (SPN_COX_) bearing COX-1/2 binder linked to a VHL-recruiting peptide. SPN_COX_ is activated by cathepsin B overexpression specific to the tumor microenvironment (Figure 2). Further, the SPN_COX_ nanoparticle polymer can generate ^1^O_2_ under near-infrared photoirradiation eliminating tumor cells and leading to immunogenic cell death. This phototherapy effect synergizes with COX-2 degradation as tumor-associated antigens are released, thereby stimulating effector T cells and enhancing tumor immunogenicity. Treatment with SPN_COX_ resulted in COX-2 depletion and was rescued by NAE and proteasome inhibition, confirming the ubiquitin-proteasome degradation mechanism. The combination of SPN_COX_ with focused near-infrared irradiation led to tumor growth suppression and a survival benefit for treated and irradiated breast cancer (4T1)-bearing mice. Alongside these findings, up-regulated effector T cells and down-regulated regulatory T cells were detected with flow cytometry, confirming the immunomodulatory effect of the PROTAC SPN_COX_. In this study, COX-2 was validated as a potential target for cancer immunotherapy. Especially, the synergy of phototherapy as immune stimuli combined with the inhibition of immune suppression by COX-2 depletion was highlighted.

## Conclusion

Since many immune reactions are reliant on protein–protein interactions and immunosuppressive protein functions might extend beyond their canonical enzymatic role, the use of targeted protein degraders provides a real advantage for future cancer immunotherapy development. Furthermore, targeted protein degradation introduces changes to the immunopeptidome, which can be targeted with bi-functional T cell engagers. Future development could also include specific covalent functionalization of presented peptides (neo-haptenization) to induce and stabilize complexes with antibodies or TCRs for augmented immune reactions.

**Figure 3:**
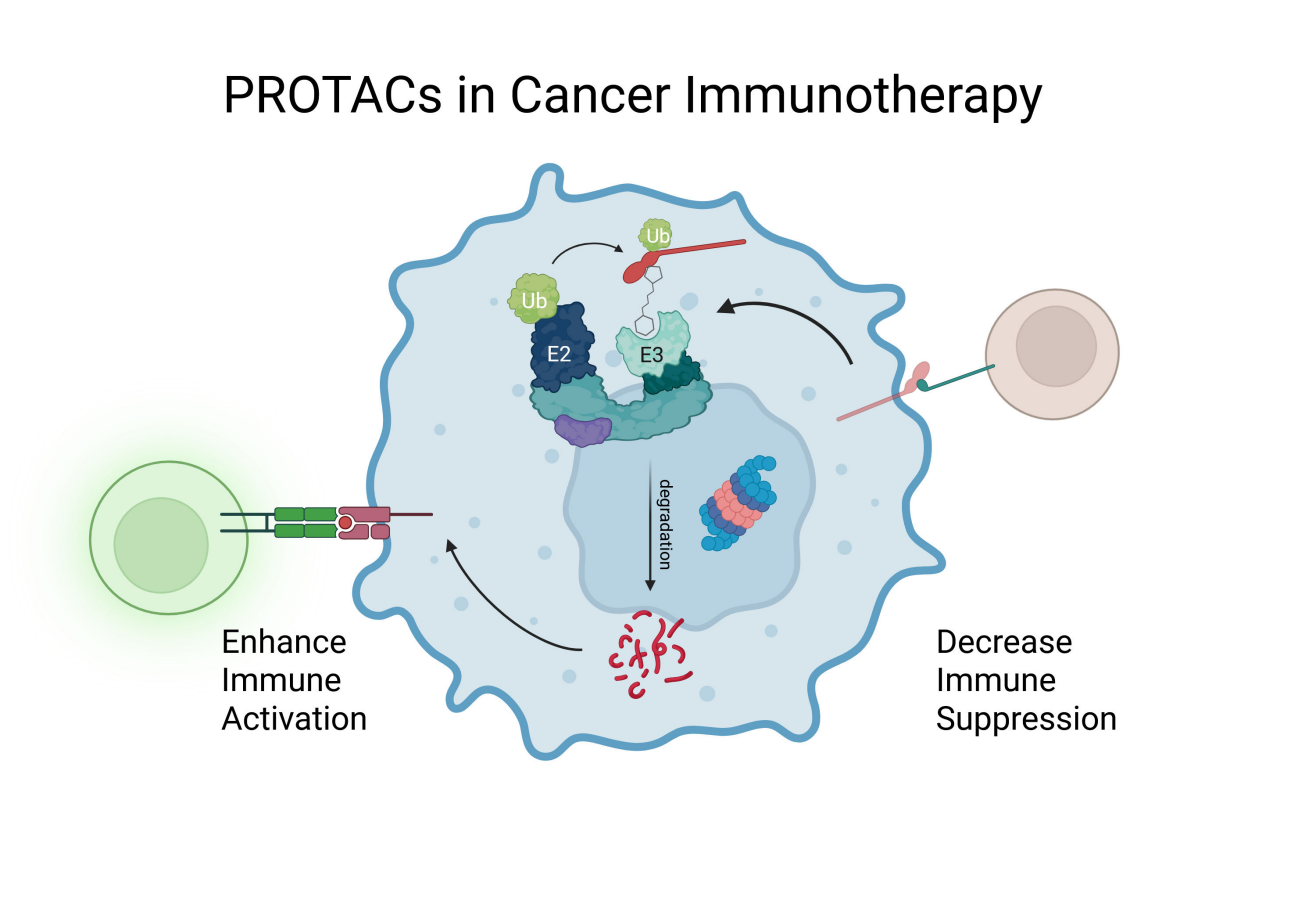
Schematic illustration of how the degradation character of PROTACs can dually benefit immunotherapy through proteolysis of immunosuppressive proteins and neoantigen creation. Figure created with Biorender.

We summarize preclinical advances in PROTAC development against promising immunosuppressive targets and their advantages in immunotherapy. However, advancing such strategies into *in vivo* and clinical pipelines as part of novel therapeutic options to increase the response to ICB therapies faces challenges. PROTACs have distinct characteristics from traditional small molecules, including ADME properties and clinical risk profiles. Some challenges include inherent toxicity of certain constituents (e.g. thalidomide), varying effects based on audience and application, high and non-specific binding as well as solubility. Thus, establishing a framework and guidelines for developing and testing PROTACs should be based on *in silico*, *in vitro*, and *in vivo* methods is a potential solution [55,56]. Next-generation PROTACs mitigate toxicity with novel CRBN binders excluding the molecular glue activities [57] and solubility as well as ADME issues with highly ordered rigid linkers and thorough lead optimization [56,58]. These optimizations even enable orally bioavailable PROTACs [59,60]. Though their large molecule sizes compared with traditional small molecule drugs, PROTACs are an exciting new therapeutic modality soon to enter the market and could show great benefit in overcoming some of the big challenges in immunotherapy and overcoming ICB therapy failure (Figure 3).

**Perspectives**

- Targeted protein degradation as well as immunotherapy are fast-developing fields of high interest in cancer drug discovery. The promising preclinical data described here and elsewhere suggest that their combination in future clinical investigations could lead to new treatment options for non-responding cancer patients.
- PROTACs offer exciting new possibilities in immuno-oncology ranging from new targets to totally new mechanisms of action. However, clinical advancement can be challenging due to pharmacokinetic restrictions and needs to be carefully investigated.
- New and highly advanced molecular designs of PROTACs could facilitate moving some of these molecules into the clinic, where finally their effectiveness will be evaluated. Emerging possible combination treatments for ICB non-responder patients represent the most exciting avenues for future investigation.

## Abbreviations

ADME: Absorption, Distribution, Metabolism, Excretion
B2M: Beta-2 Microglobulin
CIR: covalent immune recruiters
COX-1/2: cyclooxygenase-1/2
CRN: cereblon
CuAAC: copper-assisted alkyne-azide cycloaddition
DRiPs: defective ribosomal products
ER: endoplasmic reticulum
ICB: immune checkpoint blockade
IDO-1: indoleamine 2,3-dioxygenase 1
IFNγ: interferon γ
MHC-I: major histocompatibility complex I
PD-L1: programmed death ligand 1
PGE2: prostaglandin E2
POI: protein of interest
PROTACs: proteolysis-targeting chimeras
TCRs: T cell receptors
VHL: von hippel lindau
WT1: Wilms tumor protein 1.
